# Disordered eating in diet-treated inborn errors of metabolism: screening and behavioral insights

**DOI:** 10.1186/s13023-026-04420-4

**Published:** 2026-06-13

**Authors:** Mary Kate LoPiccolo, Claire Cinnamon, Colleen Donnelly, Ilona Ginevic, Jessica Waite, Sara Donnelly, Mingyu Ma, Caroline Staccone, Lissette Estrella, Alan Weinberg, Thomas Hildebrandt, Margo Sheck Breilyn

**Affiliations:** 1 Department of Genetics and Genomic Sciences, Icahn School of Medicine at Mount Sinai, New York, NY USA; 2 Department of Population Health Science and Policy, Icahn School of Medicine at Mount Sinai, New York, NY USA; 3 Department of Psychiatry, Icahn School of Medicine at Mount Sinai, New York, NY USA

**Keywords:** Inborn errors of metabolism, Eating disorder, Nutrition, Screening, Comorbidities

## Abstract

**Background:**

Lifelong restrictive diets are a mainstay of management for many inborn errors of metabolism (IEMs). The experience of metabolic care providers suggests higher prevalence of disordered eating behaviors in this population; however, this has not been well characterized. In this study, we applied screening tools adapted to our population to identify eating disorders and characterize associated symptomatology. All participants were given 2 assessment tools: the SCOFF questionnaire, a 5-question screen for a possible eating disorder, and a modified Eating Pathology Symptoms Inventory (EPSI), a 45-item Likert-scaled questionnaire characterizing eating disorder pathology. Patients aged 16 years and older on prescription diets at the Program for Inherited Metabolic Diseases at the Icahn School of Medicine at Mount Sinai were recruited. Participant demographics were collected through medical record review.

**Results:**

Fifty-two subjects were enrolled, and 51 completed both surveys. 27 subjects identified as female (52%), 23 as male (44%), and two as genderqueer (4%). Ages ranged from 16 to 50 years old. The most common diagnosis was phenylketonuria (*n* = 34, 65%), followed by glycogen storage disease type 1a (*n* = 8, 15%). Most patients were on a low-protein diet (*n* = 41, 79%), 17% (*n* = 9) were on a low-carbohydrate diet, and 6% (*n* = 3) were on a low-fat diet. The SCOFF screen positive rate was 34.6%, 95% CI [22.0% − 49.1%], which is significantly greater than population norms of 10.1% (*p* < 0.001). EPSI results revealed that our participants were significantly more likely to demonstrate binge eating, restrictive behaviors, excessive exercise, and negative attitudes towards obesity when compared to population norms.

**Conclusion:**

This study demonstrates a significantly increased screen positive rate of likely eating disorders among patients with IEM, about three times that of the general population. Metabolic providers should be aware of this potential risk to their patients, as eating disorders can impact metabolic control and adherence to diet, while also causing significant psychological distress to patients with these rare disorders. These results demonstrate that heightened awareness of potential eating disorders in patients with IEMs is warranted, and the development of validated screening tools may be impactful to their care and management.

## Introduction

Eating disorders (EDs) are life-threatening psychiatric conditions characterized by atypical eating behaviors and an impulse to control bodyweight. The major specified types of EDs described in the Diagnostic and Statistical Manual of Mental Disorders, 5th Edition (DSM-V) are anorexia nervosa, bulimia nervosa, binge eating disorder, avoidant restrictive food intake disorder, pica, and rumination disorder [1, 2]. Presentations are highly variable, and they affect individuals from all genders, races, and socioeconomic backgrounds [3, 4]. The lifetime prevalence of eating disorders is 2 to 5%, however the incidence has been rising since the onset of the COVID-19 pandemic, particularly among young adults [4]. EDs can be debilitating—psychologically and physically. Only 50% of patients fully recover from EDs, and timely diagnosis and treatment are critical in optimizing prognosis [5].

Patients with chronic diseases, particularly those managed with dietary therapy, are at high risk of developing EDs [6–8]. Mortality rates are three times higher in individuals with EDs when compared to the general population, so when considered in the context of a comorbid chronic disease, EDs are exceedingly dangerous [9]. While medically necessary, the rigorous dietary restrictions prescribed in chronic diseases, such as Type 1 diabetes, cystic fibrosis, inflammatory bowel disease, and inborn errors of metabolism, may encourage destructive relationships with food and dangerous eating behaviors [6–8].

Dietary therapy is one of the cornerstones of management in inborn errors of metabolism (IEMs) [10]. To mitigate the impact of enzymatic deficiencies and the resulting accumulation of detrimental metabolites, strict restriction of macronutrients, such as protein, carbohydrates, or fat, is prescribed. Patients must precisely measure their food intake, limit their food choices, and supplement their diets with medical foods and formulas to meet minimum nutritional requirements. Although early and consistent dietary control are a critical part of optimizing outcomes, long-term dietary adherence is very difficult to achieve. A study on patients with PKU revealed that 73% of both adults with PKU and caregivers found maintaining the diet challenging [11] Participants recognized it as a daily burden, demanding significant discipline and self-control. This study also showed that 55% of PKU patients had difficulty controlling their weight, and 14% had an ED or exhibited disordered eating. Only 4% were receiving therapy for EDs [11].

Research on the prevalence and characterization of eating disorders among patients with IEMs is limited. A retrospective cohort study of 3,714 patients with phenylketonuria (PKU) screened for comorbid neuropsychiatric conditions based on ICD-9 codes noted significantly increased prevalence of eating disorders (3.4%) when compared to the general population (0.9%) and patients with another chronic, diet-managed condition, Type 1 diabetes mellitus (1.9%) [12]. Another study examined eating behaviors and attitudes of patients with PKU by administering a survey loosely adapted from the Eating Attitudes Test (EAT-26) [13]. This study found that patients with poor metabolic control showed a higher prevalence of disordered eating behaviors, though it acknowledged the limited utility of the data given the small sample size (*n* = 15). One study examined eating behaviors and attitudes in 64 patients with hepatic glycogen storage diseases using the Eating Disorders Inventory (EDI-3), EAT-26, and Body Esteem Scale (BES), along with the corresponding pediatric versions. Participants reported increased abnormal attitudes towards food and eating and lower body esteem [14]. However, they also reported fewer classic symptoms of eating disorders, suggesting that tools assessing for eating disorders among GSD patients may need to be specifically tailored to the patient population. Importantly, discerning adaptive treatment behaviors versus true psychopathology can be difficult in patients with IEM prescribed rigorous dietary control. This presents a critical limitation in studies administering validated ED screening tools to the IEM population; however, insights collected remain valuable in a field that has yet to be thoroughly explored.

This study screened patients with IEMs treated with dietary management for eating disorders and characterized their symptoms. We accomplished this by offering participants two questionnaires, the SCOFF survey and a modified Eating Pathology Symptoms Inventory (EPSI), and by collecting individual patient perspectives.

## Methods

### Participants

**Fig. 1:**
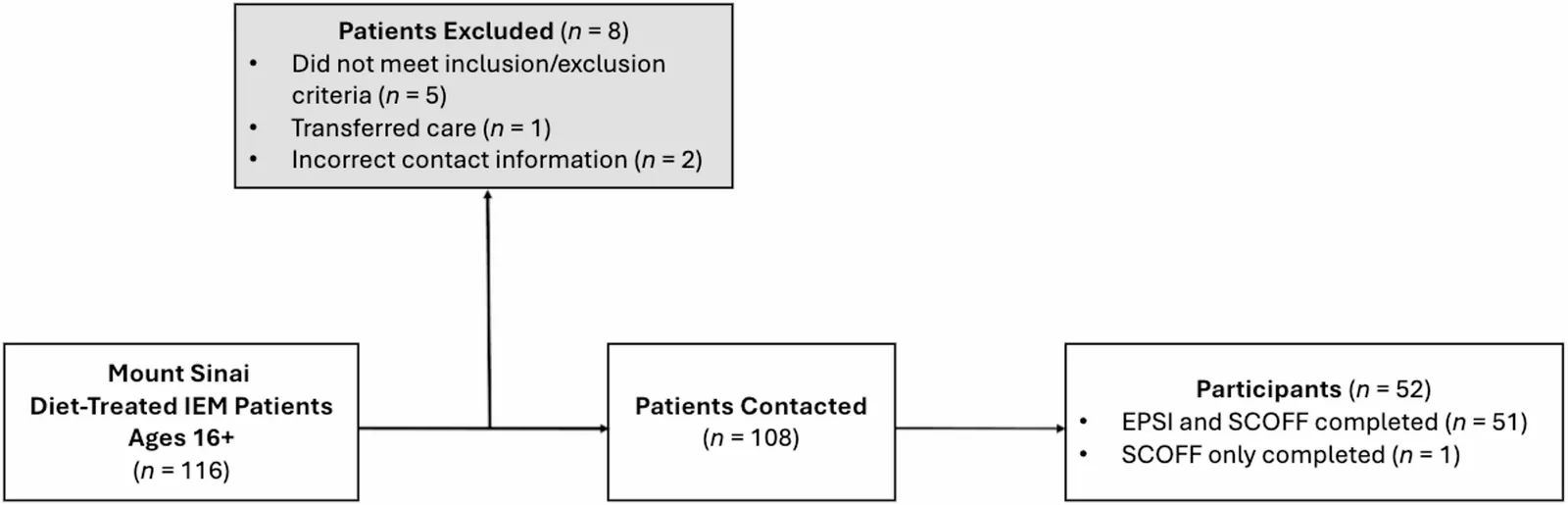
Participant recruitment

Study participants were recruited from the Mount Sinai Program for Inherited Metabolic Diseases in New York, New York, USA between October 2024 and January 2025. Eligible patients were actively followed by the clinic, had an IEM managed with diet, and were 16 years of age or older. Patients with intellectual disability impairing their ability to consent and/or comprehend the study questionnaires, pregnant patients, non-English speakers, and patients perceived to be unfit to participate by the study team were excluded (*n* = 5). Two additional patients were excluded due to incorrect contact information listed, and one was excluded due to transfer of care. 108 patients were contacted for recruitment, with 52 patients participating. 51 patients completed both the EPSI and SCOFF surveys, and one patient only completed the SCOFF survey (Fig. 1). Medical records were reviewed to determine participant eligibility and to collect patient demographics following the consenting process. Demographic information included age, sex assigned at birth, gender identity, height, weight, BMI, race/ethnicity, metabolic disorder, diet prescription, ICD-10 codes, and concomitant medications.

### Procedure

All study procedures were approved by the Mount Sinai Institutional Review Board (IRB 21-01385). Recruitment was conducted by approaching eligible participants in the clinic with a flyer or remotely with telephone calls and emails. Participants or legal guardians were prompted to review and e-sign the consent form. An assent form was required for minors. Participants were prompted to answer questions for 2 questionnaires (see “Assessment Tools” below). An optional open-ended text box was presented for additional comments in response to the questionnaires. After completion, participants were directed to an optional resources and referrals page, which gave contact information for the Mount Sinai Eating and Weight Disorders Program, and they were emailed a $10 Amazon gift card for their time.

## Assessment tools

### SCOFF questionnaire

The SCOFF questionnaire is a self-report screening tool for eating disorders, consisting of five “yes” or “no” questions [15, 16]. “SCOFF” is an acronym for the 5 questions asked, remembered with the mnemonic “Sick, Control, One-Stone, Fat, Food.” For example, “S” represents the word “sick” in the question, “Do you make yourself sick because you feel uncomfortably full?” Other items inquire about loss of control and overeating, weight loss, feeling fat, and feelings of a food-dominated life. Two or more “yes” answers qualify as a positive screening.

### The Eating Pathology Symptoms Inventory (EPSI)

The Eating Pathology Symptoms Inventory (EPSI) is a self-report questionnaire used to characterize eating disorder pathology [17, 18] The EPSI is composed of 45 statements. Participants rate each using a 5-point Likert scale (0 = Never, 1 = Rarely, 2 = Sometimes, 3 = Often, 4 = Very Often) to report how often the statement applied to them over the preceding 4 weeks. The EPSI contains 8 subscales: Body Dissatisfaction, Binge Eating, Cognitive Restraint, Purging, Restriction, Excessive Exercise, Negative Attitudes towards Obesity, and Muscle Building. Each subscale has a different number of corresponding items, ranging from 3 to 8, resulting in total possible scores ranging from 12 to 32 among subscales. A modified EPSI was utilized for the purpose of the IEM patient population, changing three items:

(1) “I used protein supplements” became “I used protein supplements *that were not prescribed to me*,” (2) “I made myself vomit in order to lose weight” became “I made myself vomit in order to lose weight *or to adhere to my metabolic diet*,” and (3) “I used muscle building supplements” became “I used muscle building supplements *that were not prescribed to me*.”

### Data analysis

To explore potential associations among the variables including age, gender, BMI, EPSI sub-scale, metabolic diagnosis, and a positive screening on the SCOFF survey, we used logistic regression modeling. Variables were first analyzed univariately by the student t-test for continuous variables and Fisher’s exact test for discrete data. For the multivariable analysis, variables with a *p* value < 0.25 were tested in a logistic regression analysis model. Odds ratios and associated 95% confidence intervals were then generated by exponentiating the beta coefficients (slope estimates) from this equation. A Hosmer and Lemeshow goodness of fit test was performed. When comparing sample means to a known population parameter, a one sample t-test was used. For all statistical analysis, data was analyzed using the SAS System software 9.4 (SAS Institute, Inc., Cary, N.C.).

## Results

### Demographics

Fifty-two patients were enrolled in the study. Of the 52 subjects, 51 subjects completed both the SCOFF and the EPSI questionnaires; one participant only completed the SCOFF. Nineteen of the participants filled out responses in the optional open-ended text box. Participant demographics are shown in Table 1. Twenty-seven participants identified as female (51.9%), twenty-three as male (44.2%), and two identified as gender queer (3.8%). There were nine IEMs represented in the study population. Most participants had phenylketonuria, or PKU (*n* = 34, 65.4%). Other represented IEMs included glycogen storage disease type 1a, or GSD1a (*n* = 8, 15.4%), maple syrup urine disease, or MSUD (*n* = 3, 5.8%), ornithine transcarbamylase deficiency, or OTC (*n* = 2, 3.8%), homocystinuria (*n* = 1, 1.9%), multiple acyl-Coa dehydrogenase deficiency (*n* = 1, 1.9%), Citrin deficiency (*n* = 1, 1.9%), carbamoyl phosphate synthetase I deficiency, or CPS I (*n* = 1, 1.9%), and carnitine palmitoyltransferase II deficiency, or CPT 2 (*n* = 1, 1.9%). Of the participants, only 3.8% (*n* = 2) were minors aged 16–17 years, while the majority were adults under the age of 50 years old (*n* = 49, 94.2%). One participant was over 50 years old (*n* = 1, 1.9%). The mean age was 29 years. Participant BMI ranged from 16.5 to 40.9 kg/m^2^, with a mean BMI of 27.5 kg/m^2^. Based on CDC BMI classifications, 3.8% (*n* = 2) of the participants were underweight, 38.5% (*n* = 20) were normal weight, 57.7% were overweight or obese (*n* = 30). Most participants were on a protein-restricted diet (*n* = 41, 78.8%), while 17.3% (*n* = 9) were on a low-carbohydrate diet and 5.8% (*n* = 3) were on a fat-restricted diet. Some patients were on a combination of these dietary prescriptions. More than half of participants (*n* = 29, 55.8%) used both medical food and formula, while 30.8% (*n* = 16) only used medical formula. On review of ICD10 codes, one individual had a pre-existing diagnosis of bulimia nervosa and another had a diagnosis of anorexia nervosa.

### SCOFF results

The overall SCOFF screen positive rate was 34.6% (*n* = 18), 95% CI [22.0%, 49.1%]. This indicates that over a third of the participants responded ‘yes’ to two or more SCOFF questions and were identified as having a likely eating disorder. Of the male participants, 26.1% (*n* = 6) screened positive, while 37.0% (*n* = 10) of the females and 100% *(n =* 2) of those identifying as gender queer screened positive (see Table 2). The average age of those who screened positive was 28.6 years old, and the average BMI was 29.6 kg/m^2^. There was no statistically significant difference in screen positive rates based on sex assigned at birth, gender identity, age, or BMI.

Of the five SCOFF items, nearly half (*n* = 24, 46.2%) of participants responded ‘yes’ to the item, “Would you say food dominates your life?” The question with the next highest response rate was, “Do you worry you have lost control over how much you eat?” with 30.8% (*n* = 16) responding ‘yes’. The complete SCOFF item breakdown is seen in Table 3. Both individuals with preexisting diagnoses of eating disorders screened positive on the SCOFF.

Analysis of SCOFF screen–positive rates by individual disorder was conducted (Table 4). Thirteen of the 34 individuals with PKU screened positive on the SCOFF, compared with 1 of 2 with OTC deficiency, 3 of 8 with GSD1a, and 1 of 1 with homocystinuria. All other individuals screened negative. Sub-analysis for individual diseases was not conducted due to insufficient power.

The SCOFF screen positive rate among our subjects was found to be significantly greater (*p* < 0.001) than the reported population norm, which is about 10% among a multiethnic UK community sample [16].

### EPSI results

The mean score for each of the eight EPSI subscales was calculated for the study population. Study population EPSI characteristics, including number of items per subscale, total possible score, score range, mean, standard deviation, and 95% confidence intervals are presented in Table 5. The only subscale where a participant responded, “very often” (i.e., a score of 4) for each item was restricting.

**Fig. 2:**
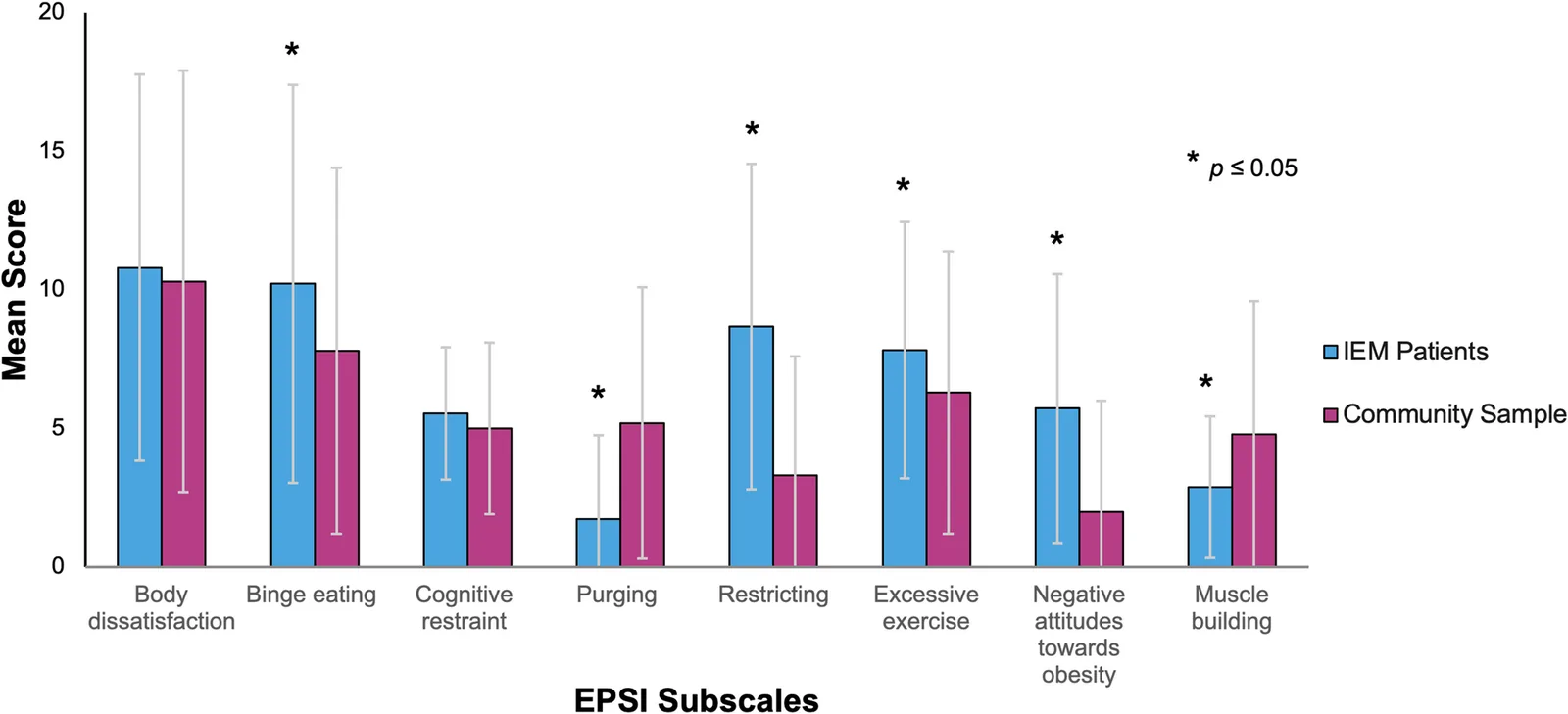
EPSI subscale scores in study population compared to community sample [18]. Legend: EPSI mean subscale scores among study population were compared to a community sample. IEM patients managed with diet were found to have significantly higher mean scores for binge eating, restricting, excessive exercise, and negative attitudes towards obesity. They had significantly lower scores for purging and muscle building behaviors

Based on multiple logistic regression for screen positivity (goodness of fit 0.53), SCOFF positivity was associated with binge eating (OR = 1.24, *p* = 0.00) and cognitive restraint (OR = 1.50, *p* = 0.05). When compared to population norms, study subjects were significantly more likely to demonstrate binge eating, restrictive behaviors, excessive exercise, and negative attitudes towards obesity [18]. Study subjects were significantly less likely to report purging and muscle building behaviors. (Fig. 2; Table 5).

The open-ended text box responses provided anecdotal commentary related to eating disorder symptom pathology as well. Patterns of restriction were seen by several participants. One noted, “At one point as a teenager I stopped eating food entirely, only surviving on the protein replacement tablets prescribed to me.” Responses explicitly mentioning purging were also seen by several participants. One stated, “I no longer make myself throw up, but I did consistently for about a year” while another participant said, “Recovered from Bulimia, used to (10 years ago) routinely purge after eating. I have related my difficult relationship with food heavily to my childhood growing up with PKU.”

## Discussion

This study screened young adult and adult patients with IEM for EDs and characterized their pathologies using two screening tools, the SCOFF and EPSI. The results suggest that there is a significantly higher screen positive rate of eating disorders among individuals with IEM treated with diet, with our population three times more likely to screen positive for an ED compared to a population norm. Study participants were significantly more likely to demonstrate binge eating, restrictive behaviors, excessive exercise, and negative attitudes towards obesity when compared to a community sample, whereas they were less likely to report purging and muscle building behaviors. Our study is the largest of its kind to-date and included patients from the widest variety of IEMs thus far in the literature.

This study has several important limitations. Our cohort size was small, particularly among individual disease groups, limiting the statistical power to detect associations between survey responses and diagnoses. Although our study population included individuals with a range of metabolic diagnoses, the majority (65.4%) had phenylketonuria. Accordingly, extrapolation of our findings to other diet-treated disorders should be undertaken with caution. Subgroup analyses of SCOFF-positive rates across disease groups were performed; although not statistically significant, they are presented to reflect the variability within the cohort.

The SCOFF is a screening tool rather than a diagnostic instrument; thus true prevalence could not be determined. In addition, despite being extensively studied in the general population, neither the SCOFF nor the EPSI have been validated in the diet-treated IEM population. Moreover, the EPSI was modified, to avoid score inaccuracies related to adaptive treatment behaviors. Although comparisons were made between the IEM population and community sample results, these are indirect, exploratory, and potentially invalidated by item modifications.

A major concern is that when screening patients with IEMs treated with diet for EDs, there is a significant risk of misclassification, as the results of screening and characterization tools may reflect necessary treatment-related behaviors rather than true underlying psychopathology. Patients with IEM treated with diet are often raised from a young age to constantly be conscious of their food intake, monitor and measure it closely, and plan their meals in advance to ensure their health. Thus, endorsement of preoccupation with food, cognitive restraint, or restrictive eating behaviors may not be indicative of an eating disorder, but rather the daily reality of living with a diet-treated IEM. It is notable that nearly half of participants indicated that food dominates their life on the SCOFF questionnaire. Similarly, the significantly higher EPSI scores for cognitive restraint and restricting may be influenced by adherence to a therapeutic diet.

These findings demonstrate that heightened awareness of potential eating disorders in patients with IEMs is warranted. The high screen positive rate on the SCOFF among individuals with IEMs managed with diet raises concern about the co-occurrence of EDs in IEMs. Further work must be done to validate the use of the SCOFF and EPSI in patients with IEMs. If this correlation between EDs in IEMs is further characterized, then future studies may include screening for pediatric patients as well, as EDs may begin to present earlier in childhood. These data suggest that maladaptive eating behaviors and related symptom endorsement may be common in some diet-treated IEM patients, and clinicians should consider careful multidisciplinary assessment.

**Table 1:** Participant demographics

	Frequency (*n* = 52)	Percentage (%)	95% Confidence Interval (%)
Age (years):			
16–17	2	3.8	0.0–9.1
18–29	23	44.2	30.7–57.7
30–49	26	50.0	36.4–63.6
50+	1	1.9	0.0–5.7
Gender			
Male	23	44.2	30.7–57.7
Female	27	51.9	38.3–65.5
Gender Queer	2	3.8	0.0–9.1
BMI			
Underweight (< 18.5 kg/m2)	2	3.8	0.0–9.1
Normal Weight (18.5–25 kg/m2)	20	38.5	25.2–51.7
Overweight (25–30 kg/m2)	14	26.9	14.9–39.0
Obesity (> 30 kg/m2)	16	30.8	18.2–43.3
Metabolic Disorder			
Glycogen storage disease type 1a	8	15.4	5.6–25.2
Maple syrup urine disease	3	5.8	0.0–12.1
Ornithine transcarbamylase deficiency	2	3.8	0.0–9.1
Phenylketonuria	34	65.4	52.5–78.3
Other*	5	9.6	1.6–17.6
Diet Prescription			
Low Protein	41	78.8	67.7–90.0
Low Fat	3	5.8	0.0–12.1
Low Carbohydrate	9	17.3	7.0–27.6
Use of Medical Food and Formula	29	55.8	42.3–69.3
Use of Medical Formula Only	16	30.8	18.2–43.3

*Carbamoyl phosphate synthetase I deficiency, carnitine palmitoyltransferase II deficiency,citrin deficiency, homocystinuria, multiple acyl-Coa dehydrogenase deficiency

**Table 2:** SCOFF screening results demographics

SCOFF Characteristics	Positive	95% Confidence Interval	Negative
	*n* (%)	%	*n* (%)
Male	6 (26.1)	8.1–44.0	17 (73.9)
Female	10 (37.0)	18.8–55.3	17 (63.0)
Gender queer	2 (100.0)	1.6–100	0 (0.0)
Total Population	18 (34.6)	22.0–49.1	34 (65.4)
	M (SD)		M (SD)
Age (years)	28.6 (9.7)	23.8–33.4	29.8 (7.9)
BMI (kg/m2)	29.6 (5.8)	26.6–32.5	26.5 (6.2)

Legend: SCOFF screen positive is defined as ≥ 2 yes responses

**Table 3:** SCOFF item breakdown and total screen positive rate

SCOFF Item Breakdown		
	*n* (%)	95% Confidence Interval (%)
S- Do you make yourself Sick because you feel uncomfortably full?	7 (13.5%)	4.2–22.7
C- Do you worry you have lost Control over how much you eat?	16 (30.8%)	18.2–43.3
O- Have you recently lost more than One stone in a three- month period?	7 (13.5%)	4.2–22.7
F- Do you believe yourself to be Fat when others say you are too thin?	13 (25.0%)	13.2–36.8
F- Would you say Food dominates your life?	24 (46.2%)	32.6–59.7
Total Screen Positive (≥ 2 yes responses)	18 (34.6%)	22.0–49.1

**Table 4:** SCOFF screen positive rate by metabolic disorder

Metabolic Disorder	Frequency (*n* = 52)	SCOFF Positive *n* (%)	95% Confidence Interval (%)
Carbamoyl phosphate synthetase 1 deficiency	1	0 (0)	0.0 - 97.5
*Carnitine palmitoyltransferase II deficiency*	1	0 (0)	0.0 - 97.5
Citrin Deficiency	1	0 (0)	0.0 - 97.5
Glycogen Storage Disease 1a	8	3 (37.5)	8.5–75.5
Homocystinuria	1	1 (100)	2.5–100.0
Multiple acyl-CoA dehydrogenase deficiency	1	0 (0)	0.0 - 97.5
Maple syrup urine disease	3	0 (0)	0.0–70.8
Ornithine transcarbamylase deficiency	2	1 (50.0)	1.3–98.7
Phenylketonuria	34	13 (38.2)	22.1–56.4
Total	52	18 (34.6)	22.0–49.1

Legend: SCOFF screen positive is defined as ≥ 2 yes responses

**Table 5:** EPSI characteristics and comparison to population norms

EPSI Characteristics							
	Number of Items	Total Possible Score	Score Range	Study Mean (SD) *n* = 51	95% Confidence Interval	Community Mean (SD) *n* = 341^18^	*p*-value
Body dissatisfaction	7	28	0–27	10.8 (6.96)	8.9–12.8	10.3 (7.6)	0.66
Binge eating	8	32	0–30	10.22 (7.17)	8.2–12.2	7.8 (6.6)	0.02
Cognitive restraint	3	12	0–10	5.55 (2.39)	4.9–6.2	5.0 (3.1)	0.23
Purging	6	24	0–12	1.73 (3.02)	0.9–2.6	5.2 (4.9)	0.00
Restricting	6	24	0–24	8.67 (5.87)	7.0–10.	3.3 (4.3)	0.00
Excessive exercise	5	20	0–17	7.82 (4.62)	6.5–9.12	6.3 (5.1)	0.05
Negative attitudes towards obesity	5	20	0–19	5.73 (4.85)	4.4–7.1	2 (4.0)	0.00
Muscle building	5	20	0–10	2.88 (2.55)	2.2–3.6	4.8 (4.8)	0.01

## Data Availability

The datasets used and analyzed during the current study are available from the corresponding author on reasonable request.
